# Assessing PLA/PBSA Films for Sustainable Packaging for Moist and Perishable Foods

**DOI:** 10.3390/polym17233093

**Published:** 2025-11-21

**Authors:** Maria-Beatrice Coltelli, Francesca Cartoni, Luca Panariello, Laura Aliotta, Vito Gigante, Andrea Lazzeri

**Affiliations:** Department of Civil and Industrial Engineering, University of Pisa, 56122 Pisa, Italy; francesca.cartoni@unipi.it (F.C.); luca.panariello@ing.unipi.it (L.P.); laura.aliotta@unipi.it (L.A.); andrea.lazzeri@unipi.it (A.L.)

**Keywords:** food packaging, biobased films, polylactic acid/polybutylene succinate-co-adipate blends, whey protein

## Abstract

The environmental impact of petroleum-based plastics has accelerated the search for sustainable alternatives in food packaging. Polylactic acid (PLA), a biobased and compostable polymer, is among the most promising candidates, yet its inherent brittleness and poor moisture barrier limit its application in high-humidity contexts such as dairy packaging. This study investigates immiscible PLA/poly(butylene succinate-co-adipate) (PBSA) blend films as potential biobased packaging materials for perishable foods. Even if these blends have been already studied, limited attention has been given to the systematic characterization of the baseline barrier properties of unmodified PLA/PBSA blends in contact with liquid dairy products. Four blend ratios (PLA/PBSA = 30/70, 40/60, 50/50, 60/40 wt%) were prepared via micro-compounding and compression molding. The films were characterized through melt flow analysis, FTIR, SEM, DSC, DMTA, and tensile testing to evaluate their thermal, morphological, and mechanical properties. Crucially, moisture barrier performance was assessed under simulated dairy conditions by sealing fresh whey at 4 °C and monitoring weight loss over 30 days. Results revealed that while tensile strength and storage modulus (E’) decreased nearly linearly with increasing PBSA content, elongation at break exhibited a non-linear trend, highlighting the complex interplay between blend morphology and mechanical behavior. The study provides a baseline understanding of neat PLA/PBSA blends in contact with liquid dairy, identifying the most promising formulations for future scale-up. These findings contribute to the development of biodegradable packaging systems tailored for refrigerated, high-moisture food applications

## 1. Introduction

The widespread use of petroleum-derived plastics in food packaging (e.g., LDPE, PP, PET) has led to significant environmental pollution due to their poor biodegradability [1]. In response, from one decade there is a growing interest in replacing these materials with renewable polymers. Among these, polylactic acid (PLA), widely recognized in the literature [2,3], is one of the most promising, being industrially produced from biomass with a robust and mature presence on the market and approved for food contact [4]. However, biopolymers and, in particular, PLA generally lag behind traditional plastics in thermo-mechanical and barrier performances, which is fundamental for being useful in food packaging [5]. For example, biopolymer-based films often exhibit lower mechanical strength, flexibility and barrier efficiency than their petrochemical counterparts [6,7]. Practically, widely used petro-based films like LDPE remain much more impermeable to water vapor and gases; indeed, high-barrier polyolefins are typically benchmark materials in packaging, and their superior oxygen and moisture resistance greatly extends the shelf life of perishables [8]. As a result, despite biodegradability and clarity, PLA-based packaging application in moist food packaging is challenged by these performance gaps. PLA films are valued for their transparency, stiffness, and adequate tensile strength and are already used (for example) in applications in direct contact with food [9,10]. Yet PLA has well-known drawbacks: it is inherently brittle and its water vapor and gas barriers are relatively poor [11]. Even high-crystallinity PLA grades cannot match the barrier of conventional plastics [12], meaning that also in the former case the water readily transmits through PLA films: this is critical for dairy products and other high-moisture foods, where controlling moisture and oxygen ingress is key to preventing spoilage [13]. For example, Holm et al. [14] stated that cheeses wrapped in PLA film experienced moisture loss nearly tenfold greater than those in the control packaging (LDPE); however, no visible surface dryness was detected until after 56 days of storage, suggesting also that PLA can perform adequately under some refrigerated conditions. Specifically, many PLA films possess good aroma and oil barrier properties but fall short on oxygen and especially moisture barriers compared to PET or oriented PS [15]. Nevertheless, in general, higher water permeability remains a limiting factor, and additional strategies (plasticizers, blends, coatings) are often required and developed to meet food packaging standards [16,17,18,19] or the achievement of multilayers and composites [20,21]. For instance, coating PLA films with whey protein or chitosan improves oxygen and water resistance. Specifically, layering whey protein onto PLA significantly lowers oxygen permeability (by introducing a dense protein network) [22]. In the dairy field, which demands strict humidity control, efforts include PLA-based antimicrobial films and laminated structures to extend the shelf life of cheese and milk [23]. As far as mechanical improvement is concerned, a common approach to improve PLA’s ductility and toughness is blending with ductile polyesters [24,25]. In particular, poly(butylene succinate-co-adipate) (PBSA) is a biodegradable aliphatic copolymer with low glass transition and good extensibility. PBSA typically has a low melting point (~90 °C) and high elongation at break [26,27]. Blending PBSA into PLA greatly enhances flexibility: studies report that adding ~40 wt% PBSA can raise PLA’s elongation from a few percent to ~200% and triple its impact resistance [28]. However, PLA/PBSA blends are essentially immiscible, so the blend morphology strongly depends on composition and processing [29]. Electron microscopy of melt-processed PLA/PBSA films consistently shows a heterogeneous two-phase structure with roughly spherical PBSA-rich domains dispersed in the PLA matrix (or vice versa) [30]. The size and distribution of these domains can be tuned by processing conditions (e.g., shear and temperature) [31], which in turn affect mechanical and barrier properties. For example, Sabalina et al. [32] found that increasing the PBSA fraction in PLA/PBSA (and PHA) blends raises the melt viscosity and elasticity significantly, consistent with larger dispersed-phase loading. In short, while PLA/PBSA blends can achieve much better toughness than neat PLA, they still inherit poor intrinsic moisture barrier performance from both components, so additional fillers or compatibilizers are often needed to meet packaging requirements. However, despite these advances, realistic shelf-life tests (e.g., monitoring moisture loss or microbial growth in packaged cheese or whey) are still relatively few in the literature. Limited attention has been given to the systematic characterization of the baseline barrier properties of unmodified PLA/PBSA blends in contact with liquid dairy products. The novelty of this study is the addressing of the gap by focusing on this specific application and providing a comprehensive evaluation of the structure–property–process relationships, and by recognizing that each processing method imparts distinct structural and morphological features that influence the resulting thermomechanical behavior. Furthermore, although PLA/PBSA blends have been widely studied, their application as barrier films for liquid dairy packaging, particularly whey, remains scarcely explored and is the focus of this investigation. To this end, the films were produced at lab scale and subsequently tested as packaging materials for whey. Specifically, in the present paper, the analysis of immiscible PLA/PBSA blend films as a biobased packaging material for perishable foods has been investigated. Four blend ratios were prepared (PLA/PBSA = 30/70, 40/60, 50/50, 60/40 by weight) via micro-compounding and compression molding. These films were characterized by melt flow, FTIR, SEM, DSC, DMTA, and tensile testing to establish their thermal, morphological, and mechanical profiles. Crucially, a real environment moisture barrier performance under simulated dairy conditions was accomplished by sealing each film with fresh whey (a dairy byproduct) at 4 °C and tracking weight change over 30 days. This method mimics the high-moisture environment of refrigerated milk products. Repeated trials showed excellent reproducibility (<0.3% variation). The goal is to provide a baseline of how PLA/PBSA blends perform as perishable-food films for identifying the most promising formulations.

## 2. Materials and Methods

### 2.1. Materials

The industrially compostable polylactic acid (PLA) utilized in this study was Luminy LX175, produced by Total Corbion in Rayong, Thailand, having a MFR of 3 at 190 °C (2.16 kg, ISO 1133-2 [33]).

The industrially and home-compostable polybutylene succinate adipate (PBSA) employed was BioPBS FD92PM from Mitsubishi, based in Tokyo, Japan, having a MFR of 4 at 190 °C (2.16 kg, ISO 1133-2 [33]).

The serum was collected at Fruzzetti company (Cascina, PI, Italy) and stored in a refrigerator at 4 °C for the duration necessary to complete the studies.

### 2.2. Preparation of Blends and Films

The polymer blends with the compositions showed in Table 1 were produced using a Haake Minilab II micro-compounder (Thermo Scientific Haake GmbH, Karlsruhe, Germany). After feeding the materials into the chamber, the molten mixture was circulated in a closed loop (with the valve shut) for one minute, driven by rotating screws. The mixing was carried out at a speed of 100 rpm and a temperature of 190 °C. During mixing, torque values were recorded, representing the momentum of the forces required to mix the material. These values are directly proportional with the viscosity of the polymer melt [34]: at a constant rotational speed, higher torque indicates higher viscosity.

**Figure 1:**
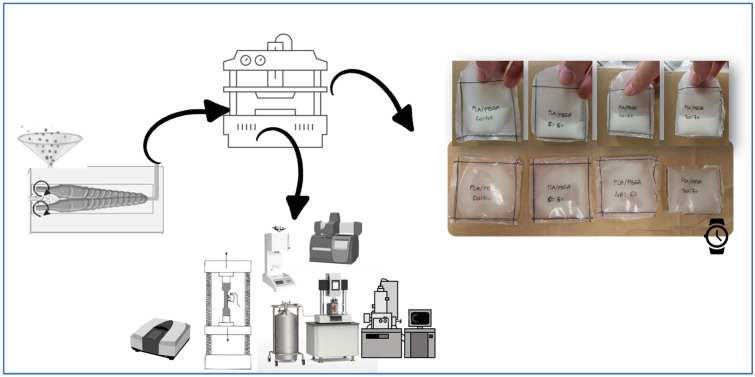
Processing (microcompounder and compression molder) and characterization equipment (DSC, MFI, SEM, DMTA, and tensile) have been showed to illustrate the pathway of the work.

Polymer films were produced using a hot-plate compression molding machine (Noselab, Bovisio-Masciago, MB, Italy), with the plates at 190 °C applying a force of 5 tons (approximately 5.3 bar) for a duration of 60 s. Each film was formed using 3 g of polymer material achieving films of 75 μm. Following the thermal molding process, the films were cooled by a stream of compressed air. After the production, some films were cut and used for chemical, rheological, thermal, and mechanical characterization. Other ones were sealed as demonstrated in Figure 1 to contain the whey.

### 2.3. Films Characterization

Flow behavior was analyzed using a CEAST Melt Flow Tester M20 (Instron, Canton, MA, USA) equipped with an encoder. The procedure followed the ISO 1133-2 D [33] at 190 °C under a load of 2.160 kg. Melt volume rate (MVR) was recorded via the encoder, while melt flow rate (MFR) was determined by weighing the extruded material. Standard deviation values for MFR were estimated by multiplying the standard deviation of MVR by the melt density (MFR/MVR).

Infrared spectra were obtained in the 550–4000 cm^−1^ range using a Nicolet 380 Fourier Transform Infrared (FTIR) Spectrometer (Thermo Fisher Scientific, Waltham, MA, USA), equipped with a Smart iTX ATR (attenuated total reflection) accessory featuring a diamond crystal. Each spectrum was collected by averaging 256 scans at a resolution of 4 cm^−1^. The ONMIC software version 9.12 was employed to adjust spectral intensities and apply spectral shifts, enabling normalization relative to selected reference bands for improved comparison of spectral profiles.

SEM micrographs were obtained using a field-emission microscope FEI Quanta 450 FEG (Thermo Fisher Scientific, Hillsboro, OR, USA) operated at an accelerating voltage of 3 kV and a beam current of approximately 50 pA. Cryo-fractured film surfaces were sputter-coated with a thin gold layer (~8 nm) prior to observation to minimize charging effects.

The thermal behavior of PLA/PBSA blend systems was characterized via differential scanning calorimetry (DSC) using a Q200 TA instrument. All measurements were conducted under a nitrogen atmosphere with a constant flow rate of 50 mL/min serving as the purge gas. Specimens for DSC analysis were extracted from film samples, with each weighing no more than 11 mg. To assess the influence of the secondary processing on the mechanical performance, the thermal analysis focused exclusively on the first heating cycle. Sampling was consistently performed from identical regions within the specimens (in the middle) to minimize variability due to differential cooling rates across the sample thickness. Prior to analysis, each sample was hermetically sealed in aluminum pans. The thermal protocol involved rapid cooling from ambient temperature to −40 °C, where samples were held for 1 min. Subsequently, heating was applied at a rate of 10 °C/min up to a final temperature of 190 °C.

Quasi-static tensile tests were conducted on rectangular specimens with a width of 10 mm and thickness of 70 μm, avoiding defects such as bubbles. A clamps distance of 50 mm was used, with 20 mm clamped at each end. Testing was performed using an INSTRON 5500R machine (INSTRON, Norwood, MA, USA) equipped with a 100 N load cell and pneumatic clamps, at a speed of 10 mm/min. Specimens were conditioned for at least 48 h at room temperature and 50% relative humidity. Five specimens per sample were tested and from this analysis stress at break and elongation at break were evaluated.

In order to accomplish a proper evaluation of the stiffness of the films, a DMA analysis (GABO Eplexor, Netszch, Selb, Germany) in tensile configuration has been carried out on three repeated films specimens with dimensions 40 mm × 10 mm and with a clamp length of 20 mm. The frequency used was 1 Hz and a temperature of 25 °C. The storage module (E’) has been evaluated.

For barrier property evaluation, two films per formulation (13–14 cm diameter) were sealed on three sides using a Cocoon packaging sealer (Cocoon S.r.L., Lissone, Italy). Fresh whey (provided by Fruzzetti Dairy, Cascina, PI, Italy) was added before final sealing. Samples were stored at 4 °C, and their weight was monitored at least over 30 days using an analytical balance.

## 3. Results

### 3.1. Melt Flow Rate Results

As shown in Table 2, the PLA/PBSA blends with all the compositions of 60/40, 50/50, 40/60, and 30/70 exhibited MFR values exceeding those of both pure components. Indeed, in the literature and in technical datasheets MFR values of the same grades of neat poly(lactic acid) (PLA) were measured at 2 g/10 min and neat poly(butylene succinate-co-adipate) (PBSA) was slightly lower, at 1.5 g/10 min at the same temperature of testing [28,35]. The existence of a MFR maximum in the blends (that corresponds to a viscosity minimum) can be explained with morphology considering that one component remains as a droplet within the matrix constituted by the other polymer. This dispersed phase is deformable during the flow, resulting in a lower shear viscosity of the blends compared to the pure components [36,37].

Going into detail, the measured melt-flow values show a clear and systematic decrease in flowability with increasing PBSA content. Both MVR and MFR diminish monotonically from the PLA/PBSA 60/40 composition to PLA/PBSA 30/70 (MVR: 4.4 → 3.5 cm^3^/10 min; MFR: 5.0 → 3.7 g/10 min). Expressed relative to the 60/40 formulation, the 30/70 blend exhibits a ≈20.5% reduction in MVR and ≈26.0% reduction in MFR. On average, increasing PBSA by 10 wt.% corresponds to a decrease of approximately 6.8% in MVR and ≈8.7% in MFR over the composition range studied. These trends indicate that PBSA-rich blends have a higher melt viscosity and lower flowability: the evolving morphology affects macroscopic flow. PBSA forms a discrete or co-continuous dispersed phase within the PLA matrix depending on composition, as will be shown in the SEM results; the presence of dispersed rubbery droplets or an interpenetrating domain structure can increase melt elasticity and hinder flow through the capillary die used [38]. Morphology-induced flow resistance is consistent with the observed decrease in flow rates as PBSA content increases.

Standard deviations are small in absolute terms but increase relatively at higher PBSA contents; this growing scatter suggests that blends with higher PBSA loading may develop more heterogeneous melt structures, and it shows minor variations in suffering processing and sample preparation, which in turn affects the melt-flow measurement.

### 3.2. Phase Morphology Evolution and Composition Effects

**Figure 2:**
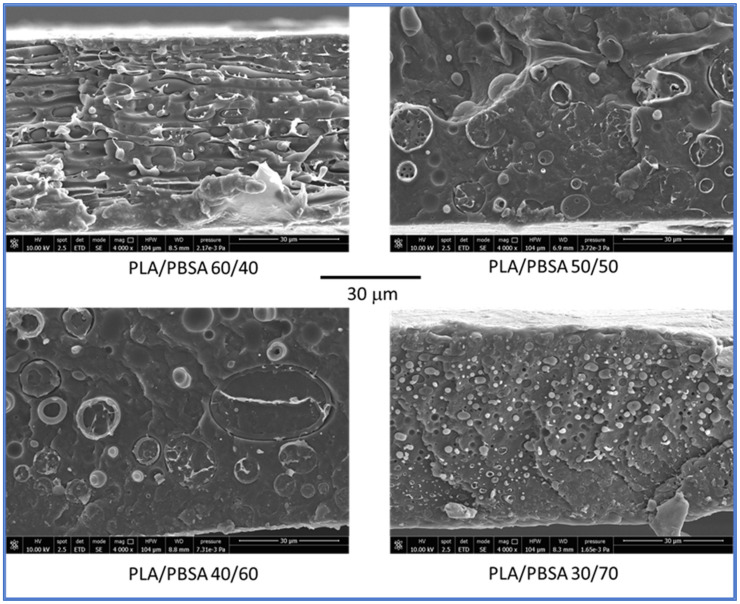
SEM micrographs obtained on cryofractured films of blends having different contents of PBSA. All the micrographs have a magnification of 4000×.

Interestingly, it is necessary to underline the processing behavior with the morphology achieved. The morphology of the blend films was examined by scanning electron microscopy as described in methodology section. For the PLA/PBSA 60/40 film, the two polymer phases exhibited a co-continuous distribution, appearing interpenetrated and predominantly oriented parallel to the plane of the film (Figure 2). A similar structure was observed in the PLA/PBSA 50/50 blend; however, the phase domains appeared larger, and no orientation of the phases was present. With a further increase in PBSA content (PLA/PBSA 40/60), the phases became noticeably coarser, and the distinction between the matrix and the dispersed phase became more apparent, but suggesting the occurrence of some detachment of the PLA rigid phase from the major PBSA matrix. This will be of fundamental importance for mechanical properties.

In the PLA/PBSA 30/70 blend, a complete phase inversion was evident, with the dispersed phase (PLA) forming micrometric domains. This composition can therefore be classified as a dispersed morphology, with PLA present as nearly spherical domains embedded within the PBSA matrix.

**Figure 3:**
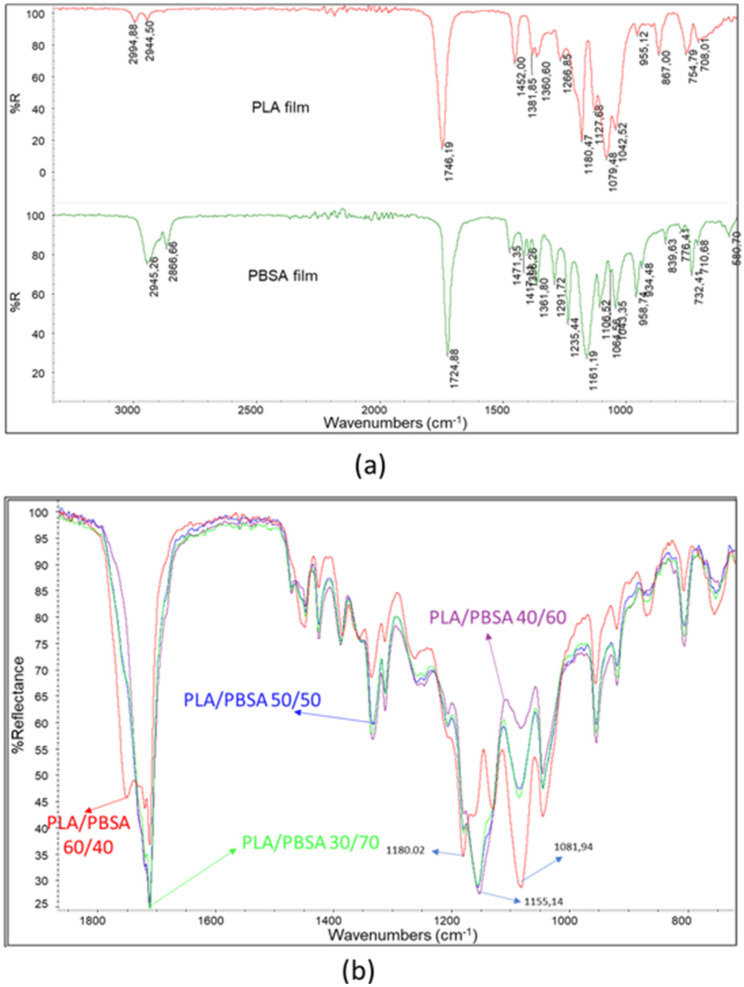
(**a**) Infrared ATR spectra of PLA and PBSA; (**b**) superposition of the spectra of the blends with different PBSA content in the range 700–1900 cm^−1^.

In order to study their surficial homogeneity, the films were characterized by infrared spectroscopy using an ATR accessory. Thanks to this technique the penetration of the infrared ray in the film is of a few microns, so the composition of the blend close to the surface can be explored. ATR spectra were recorded onto films of pure polymers (Figure 3a), and it was possible to observe that the typical stretching of C-H bonds in the 2850–2990 cm^−1^ range and C=O bonds in the 1700–1760 cm^−1^ range results were close but different. In particular, the peak maximum relative to C=O stretching for PLA is 1746.19 and for PBSA is 1724.88 cm^−1^. The fingerprint region of the spectra is also different, and the band typical of PLA has maxima wavenumbers at 1079.48 cm^−1^ and 1180.47 cm^−1^ (C-O-C stretching). On the other hand, PBSA has a main peak at 1161 cm^−1^ attributable to asymmetrical C-C-O stretching. Interestingly, Harder et al. noticed that the stretching C=O peak of PBSA was at 1712 cm^−1^, whereas the film of PBSA showed a maximum at 1724 cm^−1^. However, it should be considered that PBSA is a copolymer.

To assess the surface homogeneity of the films, attenuated total reflectance Fourier-transform infrared (ATR-FTIR) spectroscopy was employed. This technique enables the analysis of the chemical composition within the upper microns of the film, thereby providing insight into the near-surface structure of the polymer blends. ATR spectra were acquired for films composed of pure polymers, revealing distinct spectral features. The characteristic C–H stretching vibrations were observed in the 2850–2990 cm^−1^ range, while the C=O stretching bands appeared between 1700 and 1760 cm^−1^, with subtle but notable differences between the polymers. Specifically, the C=O stretching peak for poly(lactic acid) (PLA) was located at 1746.19 cm^−1^, whereas for poly(butylene succinate-co-adipate) (PBSA), it appeared at 1724.88 cm^−1^. Differences were also evident in the fingerprint region: PLA exhibited prominent bands at 1079.48 cm^−1^ and 1180.47 cm^−1^, corresponding to C–O–C stretching vibrations [39,40], while PBSA showed a dominant peak at 1161 cm^−1^, attributed to asymmetric C–C–O stretching [41]. Notably, Harder et al. reported a C=O stretching peak for PBSA at 1712 cm^−1^; however, in the present study, the PBSA film exhibited a maximum at 1724 cm^−1^. This discrepancy may be attributed to the copolymeric nature of PBSA, which can influence its spectral characteristics. In fact, based on the findings of Yao et al. [42] regarding poly(butylene succinate) (PBS) and those of Yan et al. [43] concerning poly(butylene adipate) (PBA), it can be inferred that the absorption peak near 1715 cm^−1^ corresponds to the crystalline regions of PBS. The band at 1722 cm^−1^ is associated with the rigid amorphous fraction (RAF) of PBS, while the peak around 1735 cm^−1^ is indicative of the mobile amorphous phase of PBS and also reflects the C=O stretching vibrations present in the PBA segments. Thus, in the pure PBSA film rapidly cooled following compression molding, the absorption peak at 1724 cm^−1^ suggests a predominantly amorphous polymer structure, as anticipated. The spectra of the blends varied with composition (Figure 3b). The PLA/PBSA 60/40 blend exhibited prominent peaks at 1081.94 cm^−1^ and 1180.02 cm^−1^, consistent with the spectrum of pure PLA. In contrast, for blends containing ≥50 wt% PBSA, the most prominent peak appeared at 1155.14 cm^−1^, which is attributable to PBSA.

**Figure 4:**
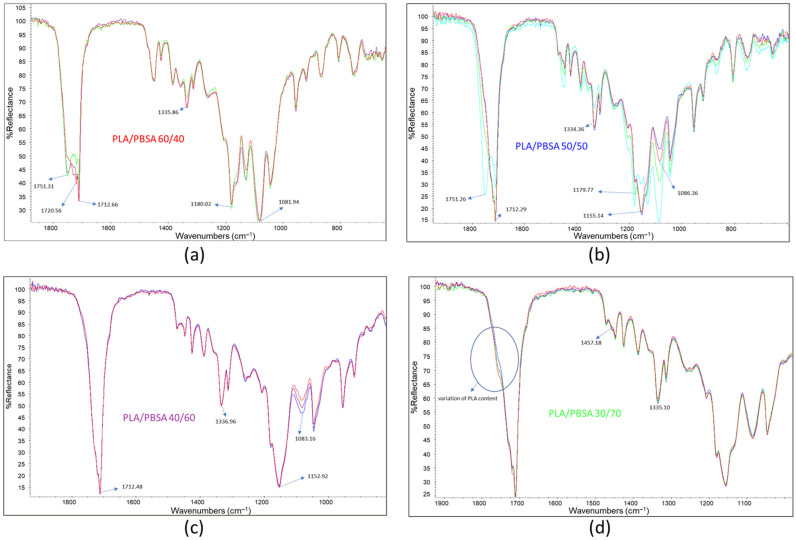
Three infrared ATR spectra overlapped: (**a**) PLA/PBSA 60/40; (**b**) PLA/PBSA 50/50; (**c**) PLA/PBSA 40/60; (**d**) PLA/PBSA 30/70.

For each film, several spectra were recorded to assess surface composition by overlapping and comparing the results. In Figure 4, three spectra are shown for each film sample. The spectrum of the PLA/PBSA 60/40 film displays C=O stretching peaks with maxima at 1751.31, 1720.56, and 1712 cm^−1^, with varying intensities across different analyzed points (Figure 4a). As previously noted, these variations can be attributed to fluctuations in surface composition, consistent with the co-continuous morphology of the blend. This morphology consists of interpenetrated phases whose local composition may vary significantly across the film. Interestingly, the most intense C=O stretching peak in the film appears at 1712.66 cm^−1^, suggesting that PBSA is predominantly crystalline [43]. It is frequently reported that in PLA/PBSA blends, PBSA crystallization can be promoted by the presence of interphases within the blend [44].

The peak at 1335.86 cm^−1^, identified by Praveena et al. [45] as indicative of the amorphous or crystalline state of PLA, also exhibits intensity variations due to fluctuations in PLA content. These variations are observable across the different spectra.

Composition fluctuations are more pronounced in the PLA/PBSA 50/50 blend film, where significant changes in peak frequency and intensity were detected (Figure 4b). In some spectra, the dominant C=O stretching peak appears at 1751.26 cm^−1^, corresponding to PLA, while in other regions the most prominent peak is at 1712.29 cm^−1^, associated with crystalline PBSA. In contrast, the spectra of the PLA/PBSA 40/60 film were highly consistent (Figure 4c), with the main C=O stretching peak centered at 1712.48 cm^−1^. This suggests that compositional fluctuations are more limited in this blend. Notably, slight variability in the intensity of the band at 1080 cm^−1^ may indicate that PLA is at least partially dispersed, allowing for minor fluctuations in its surface concentration.

The spectra of the PLA/PBSA 30/70 blend showed minimal variation (Figure 4d), indicating a high degree of surface homogeneity. The dominant peaks correspond to PBSA, although a subtle shoulder at 1750 cm^−1^—whose intensity varies slightly—suggests minor fluctuations in PLA content.

Interestingly, between the PLA-associated band at 1452 cm^−1^ and the PBSA band at 1471 cm^−1^, a shoulder at 1458 cm^−1^ is observed. This peak, typical of C–H bending vibrations, may be attributed to interactions between the C–H bonds of PLA and PBSA, which vary slightly due to limited changes in PLA surface concentration. Despite these minor variations, the overall homogeneity of this film is evident and can be linked to its specific morphology, characterized by a PBSA matrix with dispersed PLA droplets.

### 3.3. Mechanical Properties of Films

**Figure 5:**
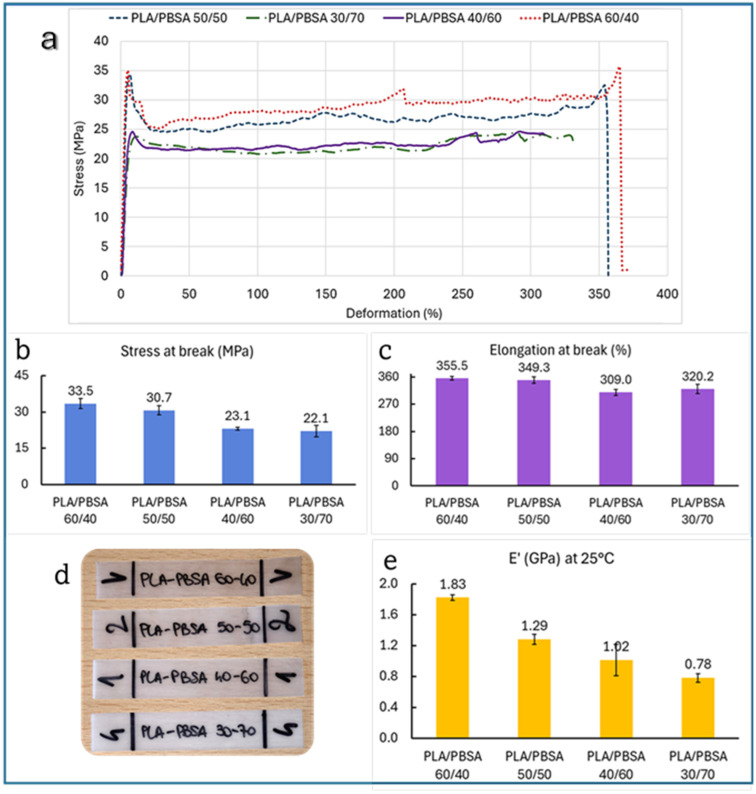
(**a**) Stress–strain curves of tested films, (**d**) images of the specimens and histograms of average data and SD of (**b**) stress at break, (**c**) elongation at break, and (**e**) storage modulus of PLA/PBSA blends.

Mechanical testing of PLA/PBSA films (Figure 5) reveals a near-linear decrease in storage modulus (E’), two different behaviors regarding the tensile stress with increasing PBSA content, while elongation at break shows a non-linear, more complex behavior.

Specifically, tensile strength drops from 35.5 MPa at 60/40 to 22.1 MPa at 30/70, and E’ decreases from 1.83 GPa to 0.78 GPa. This trend aligns with the known mechanical softness of PBSA compared to PLA, as PBSA contributes flexibility but reduces stiffness and strength when its proportion increases. This linearity is consistent with previous findings. For instance, Sabalina et al. [32] observed that increasing PBSA content in PLA/PBSA blends leads to a predictable reduction in tensile modulus and strength due to the elastomeric nature of the succinate-based polymer. Similarly, Zhao et al. reported that PLA/PBS blends show a monotonic decrease in tensile strength with increasing PBS, a polymer structurally and mechanically similar to PBSA [46]. The tensile strength data suggests the existence of two distinct performance regimes across the PLA/PBSA blend series.

Specifically, blends containing up to 50 wt% PBSA (i.e., 60/40 and 50/50) maintain relatively high tensile strength values (35.5 MPa and 30.7 MPa, respectively). However, beyond this threshold, a marked drop is observed: the 40/60 and 30/70 blends fall to 23.1 MPa and 22.1 MPa, respectively. This behavior implies the presence of a critical PBSA concentration, beyond which the structural integrity of the blend is significantly compromised. It is likely that above ~50 wt% PBSA, the PLA phase becomes too discontinuous to effectively contribute to mechanical resistance, and the mechanical response is dominated by the softer PBSA matrix. This transition aligns with the morphological shift from co-continuous to a morphology with coarser particles to finally a dispersed-phase structure, as observed in SEM micrographs.

Such “threshold behavior” has been reported in other immiscible polymer systems, where exceeding a certain volume fraction of the soft phase leads to a percolation-like loss of mechanical reinforcement [47]. Therefore, the tensile strength plateauing around 20 MPa in high-PBSA blends reflects not only compositional effects but also a morphology-driven mechanical limit.

However, elongation at break behaves differently. While one might expect a steady increase in ductility with more PBSA, the data show a non-linear trend: elongation peaks at 355.5% for the 60/40 blend, dips slightly at 50/50 (349.3%), drops further at 40/60 (309.0%), and then unexpectedly rises again at 30/70 (320.2%). This irregularity suggests that ductility is influenced not only by PBSA content but also by phase morphology, interfacial adhesion, and blend compatibility. Interestingly, as revealed by SEM analysis, the 60/40 blend exhibits a co-continuous structure, where both PLA and PBSA phases are interpenetrated with coarser particles of PLA as stated before displaying a droplet-matrix morphology. It is possible to emphasize that the co-continuous architecture of the 60/40 blend allows for more efficient stress transfer and deformation across both phases, resulting in enhanced elongation at break. In comparison, the dispersed PLA domains in the other blends act more like rigid inclusions, limiting the overall extensibility of the material. Such behavior is consistent with previous studies on immiscible polymer blends, where co-continuity is often associated with improved toughness and ductility due to synergistic deformation mechanisms [48,49,50]. Therefore, the superior ductility observed in the 60/40 blend is not solely a function of PBSA content, but also of the interconnected phase morphology, which facilitates more uniform strain distribution during tensile loading. In addition, the 40/60 blend that shows the worst elongation at break is a signal of an excessive coalescence and big dimensions of PLA droplets that cause a slight embrittlement. Moreover, such behavior has been documented in the literature. Bellon et al. [51] noted that at certain blend ratios, phase inversion or poor interfacial bonding can reduce ductility despite a higher PBSA content.

### 3.4. Thermal Behavior

The first-heating DSC thermograms of the compression-molded PLA/PBSA blend (Figure 5) films exhibit two clearly distinguishable thermal regions. The first region, extending from approximately 70 to 95 °C, is dominated by the melting and recrystallization processes of PBSA, whereas the second region, between 140 and 155 °C, corresponds to the melting of PLA [52,53]. In PBSA-rich compositions, such as the PLA/PBSA 30/70 blend, a pronounced endothermic event is observed around 87–90 °C. This feature frequently appears as multiple overlapping endotherms, consistent with the well-documented multiple-melting behavior of PBSA, which originates from the coexistence of lamellae with different thicknesses and crystallite perfection [28].

**Figure 6:**
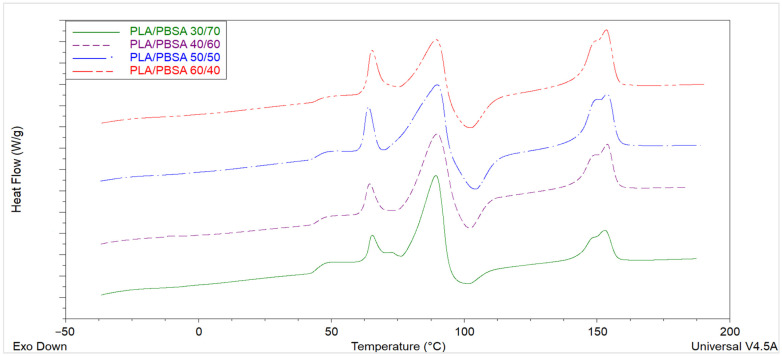
Thermograms of PLA/PBSA blends after compression molding (I run scan).

At higher temperatures, the PLA component displays a characteristic double-melting feature, typically centered around 150 °C. This double endotherm, often expressed as a shoulder followed by a main melting peak, is widely interpreted as evidence of a melt–recrystallization–remelt sequence. Imperfect or disordered α′ crystallites melt first and reorganize into more ordered α crystals, which subsequently melt at a higher temperature. Accordingly, the higher-temperature peak is attributed to the melting of more stable α-form crystals [54]. In the intermediate temperature range, an endothermic signal at approximately 65 °C can be observed, which is associated with enthalpic relaxation due to the physical aging of PLA. This feature arises from the release of stored enthalpy accumulated during secondary processing or storage below the glass transition temperature [55]. The glass transition (Tg) of PLA itself is discernible as a clear inflection in the baseline around 50 °C. Notably, the PBSA Tg remains essentially constant across the different blend compositions, indicating limited miscibility of the amorphous phases and a dominance of phase-separated morphology. Partial overlap occurs between PBSA melting, PLA cold crystallization, and enthalpic relaxation phenomena, which complicates quantitative integration of individual enthalpic contributions. PBSA melting endotherms in the 70–95 °C region may coincide with PLA cold-crystallization exotherms (typically between 70 and 120 °C) and with the aging-related endotherm near 65 °C, leading to apparent broad or asymmetric transitions. Processing history exerts a strong influence on the DSC thermograms. The cooling rate and secondary compression-molding step determine the degree of crystal perfection and the relative proportions of α′ and α crystalline forms. Overall, the thermal behavior of PLA/PBSA blends reflects a complex interplay between thermodynamic stability and kinetic constraints imposed by processing. The observed transitions—glass transitions of both components, aging relaxation of PLA, multiple PBSA melting events, and PLA’s double-melting behavior—collectively highlight the sensitivity of these biodegradable blends to their compositional ratio and thermal–mechanical history. An exothermic feature at ≈100–105 °C is observed immediately after the melting of the crystalline PBSA phase. In the literature, this peak can be consistent with either cold crystallization of PLA promoted by increased chain mobility when the secondary phase melts [56,57] or melt-recrystallization of PBSA into a more stable crystalline form [58]. Since the exothermic peak in Figure 6 becomes more pronounced with increasing PLA content, it is therefore reasonable to attribute it to the cold crystallization of PLA.

### 3.5. Barrier Properties

**Figure 7:**
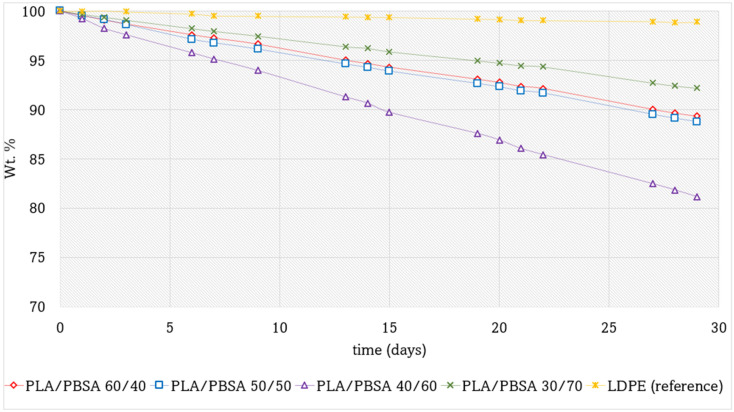
Weight percentage of perishable product as a function of time in days for the PLA/PBSA blends packaging with different PBSA content compared with a fossil LDPE film.

Figure 7 compares the mass loss curves of various PLA/PBSA film packaging formulations over time. Low-density polyethylene (LDPE) was selected as a reference material due to its well-established use in food packaging, attributed to its excellent hydrophobic properties and mechanical flexibility. The production of LDPE films followed the same pathway, and achieving the same thicknesses, of biobased films.

Among the tested blends, PLA/PBSA 30/70 exhibited the best performance in terms of moisture barrier properties, followed by PLA/PBSA 60/40 and PLA/PBSA 50/50, which showed very similar behavior. In contrast, the PLA/PBSA 40/60 blend demonstrated the highest mass loss, indicating inferior barrier performance.

These differences can be attributed to both the polarity and morphological characteristics of the blends. PBSA is inherently more apolar than PLA due to its lower content of carbonyl (C=O) groups in the polymer backbone. Consequently, the PLA/PBSA 30/70 blend, which features a PBSA-rich continuous matrix, benefits from enhanced hydrophobicity and reduced water permeability.

Morphology also plays a critical role in determining barrier properties. The PLA/PBSA 40/60 blend exhibited a coarse phase distribution with large domains and weak interfacial adhesion, as shown in Sem analysis. This poorly integrated microstructure results in an extended interfacial region with reduced density, which may facilitate water diffusion through the film. These findings highlight the importance of both chemical composition and phase morphology in designing polymer blends for effective moisture barrier applications.

Another noteworthy observation is that the trend of moisture loss over time appears to be approximately linear across all PLA/PBSA blend films, albeit with varying slopes depending on the specific composition. This suggests that while the overall kinetics of water transmission remain consistent, the rate of moisture loss is clearly influenced by the film morphology over the polymer ratio. In these systems, water is the primary migrating molecule through the film, and, therefore, the observed mass loss is closely linked to the intrinsic hydrophobicity of the materials used. Among the tested formulations, the PLA/PBSA 70/30 blend exhibited the most effective barrier properties, demonstrating the lowest weight loss and suggesting superior resistance to moisture transmission. The diffusion of humidity originating from perishable products through polymeric packaging films is a complex and multifaceted process. It is influenced not only by the intrinsic properties of the polymer, such as polarity, crystallinity, and free volume, but also by external factors like relative humidity and temperature gradients. As Turan [59] demonstrated, water vapor transport in thermoplastic polyurethane films deviates from ideal Fickian behavior under high-humidity conditions due to polymer swelling and hydrogen bonding interactions, which alter the diffusion pathways. Kumar et al. [60] further emphasized that moisture absorption in packaging films initially follows a linear trend before plateauing, suggesting saturation effects that complicate predictive modeling. Additionally, Gaikwad et al. [61] highlighted the role of moisture absorbers in mitigating excess humidity, particularly in high water activity foods, where uncontrolled diffusion can accelerate spoilage. For water vapor transmission measurements, the cumulative mass versus time curve typically becomes linear after the lag time. Recent studies have emphasized the importance of accurately determining and interpreting this initial delay to compare materials consistently and to derive reliable values of diffusivity [62]. In our case, the lag time is very short, indicating a rapid establishment of steady-state diffusion, after which the cumulative mass loss proceeds in a clear linear regime.

It could also be interesting, for a more comprehensive characterization, to carry out migration measurements. Although direct migration measurements into whey were not performed in this study due to the lack of dedicated analytical facilities, previous works have shown that PLA could release lactic acid, lactide, and low-molecular-weight oligomers under hydrolytic or storage conditions. These products are not volatile, so they could not affect the determinations carried out in this work. Moreover, Mutusuga et al. [63] described that their results showed that migration levels were relatively low at moderate temperatures (≤40 °C) and Scarfato et al. [64] underlined that a tailored structural design can control PLA-based film migration and performance. Finally, Ubeda et al. [65] stated that linear oligomers of the type CH_3_–CH_2_–O–[LA]_n_–H were not present in the films; however, their migration was observed exclusively when the simulant contained a high ethanol concentration (EtOH 95%). No significant migration occurred with simulants of lower ethanol content.

## 4. Conclusions

In conclusion, this comprehensive characterization of compression-molded PLA/PBSA films establishes a clear baseline for unmodified blend matrices intended for high-moisture food packaging. Mechanical, thermal, and rheological data show that increasing PBSA systematically reduces stiffness and strength while altering ductility in a morphology-dependent manner: a co-continuous architecture near 60/40 yields exceptional extensibility, whereas PBSA-dominant films (>50 wt%) exhibit a softer, PBSA-controlled response and a pronounced loss of reinforcement. ATR-FTIR surface analysis complements SEM and DSC findings by revealing composition-dependent near-surface heterogeneity: co-continuous and 50/50 films display spatially varying C=O signatures (PLA vs. PBSA), whereas 40/60 and 30/70 films are spectrally more homogeneous and PBSA-rich at the surface. Critically, moisture-migration tests with fresh whey at 4 °C show a very short lag time followed by a sustained linear permeation regime, indicating rapid attainment of steady-state transport with results not so far from LDPE reference. Collectively, the results identify the 50–60 wt% PLA window as the most promising starting point for targeted compatibilization, surface treatments, or multilayer architectures intended to close the gap with polyolefin barrier performance while retaining biodegradability. In future scale-up efforts, after a careful migration analysis, suitable additives or compatibilizers could then be introduced to further enhance performance, but first the idea is to which neat blends are inherently closest to meeting packaging needs. This research was undertaken to support the development of fully biodegradable flexible packaging solutions capable of replacing conventional polyolefin films with refrigerated and high-moisture food applications. The systematic characterization presented here aims to guide the optimization of PLA/PBSA blends for industrial processing and end-use performance in sustainable food-packaging systems.

## Figures and Tables

**Table 1:** Composition of blends prepared through mini extrusion.

Samples	PLA (wt%)	PBSA (wt%)
PLA/PBSA 60/40	60	40
PLA/PBSA 50/50	50	50
PLA/PBSA 40/60	40	60
PLA/PBSA 30/70	30	70

**Table 2:** Melt volume rate and melt flow rate results.

Samples	MVR (cm^3^/10 min)	MFR (g/10 min)
PLA/PBSA 60/40	4.4 ± 0.1	5.0 ± 0.2
PLA/PBSA 50/50	4.1 ± 0.3	4.5 ±0.2
PLA/PBSA 40/60	3.9 ± 0.3	4.2 ± 0.3
PLA/PBSA 30/70	3.5 ± 0.3	3.7 ± 0.4

## Data Availability

The original contributions presented in the study are included in the article, further inquiries can be directed to the corresponding authors.
